# Developing and evaluating treatments for the challenges of autism and related neurodevelopmental disabilities

**DOI:** 10.1186/s11689-021-09404-y

**Published:** 2021-11-17

**Authors:** Leonard Abbeduto, Mustafa Sahin

**Affiliations:** 1 MIND Institute and Department of Psychiatry and Behavioral Sciences, University of California, Davis, Sacramento, CA USA; 2 Rosamund Stone Zander Translational Neuroscience Center, Department of Neurology, Boston Children’s Hospital, Harvard Medical School, Boston, MA USA

In this special issue of the *Journal of Neurodevelopmental Disorders* (*JND*), we continue the annual tradition of highlighting in a thematic manner the research contributions of the NICHD-funded Eunice Kennedy Shriver Intellectual and Developmental Disabilities Research Centers (IDDRCs). The thematic focus of this year’s special issue is “intervention in autism spectrum disorder (ASD) and related conditions.” The 13 papers in this collection address issues across a wide portion of the lifespan and include tests of the efficacy of specific interventions, methods for measuring treatment efficacy in terms of behavioral symptoms and underlying neurobiological mechanisms, the identification of potential new treatment targets and approaches to treatment, and frameworks for determining who should receive particular treatments and when. These papers illustrate the breadth of the translational research supported by the 15 institutions in the IDDRC network (https://www.aucd.org/ddrcportal/directory/directory.cfm) and, more importantly, the considerable progress being made toward reducing the disabilities and challenges that are faced by many autistic individuals.

Several papers in this issue focus on documenting the efficacy of specific treatments or the feasibility and relative efficacy of different approaches to implementing already empirically supported interventions. Keefer and Vasa report on the feasibility and preliminary efficacy of a group-format, telehealth-delivered cognitive-behavioral therapy (CBT) for anxiety in 4- to 6-year-olds on the autism spectrum. Anxiety disorders occur at very high rates in people on the autism spectrum, and the few interventions that do exist have not been adapted for children this young, making this line of research highly significant. Rogers et al. and Corona et al. focus on evaluating new approaches to delivering empirically validated established interventions. Rogers et al. explored the feasibility of training Part C early intervention providers to deliver at fidelity a parent-coaching, telehealth version of the Early Start Denver Model to families in low resourced communities. Corona et al. investigated the relative acceptability and efficacy of telehealth, in-person, and hybrid versions of a behavioral intervention for young children referred to Part C providers for ASD concerns. Together, the Rogers et al. and Corona et al. studies establish the promise of telehealth delivery of treatment in ways that reduce social disparities in access.

The interventions being evaluated by the authors in this issue span different populations and approaches, ranging from pharmacological to educational, from single gene disorders with severe intellectual disability to non-syndromic autism in adults without intellectual disability. In their article, Olson et al. focus on CDKL5 deficiency disorder (CDD), a genetic disorder associated with a number of phenotypes including symptoms of ASD. Rather than examine the efficacy of a specific treatment, Olson and colleagues catalog the largely biomedical treatments commonly used for various CDD symptoms, such as sleep problems and seizures, with the hope of fostering the development of more CDD-specific approaches to treatment and evaluations of their efficacy. In their article, DaWalt et al. evaluated a multi-family group psychoeducation intervention, called Working Together, for adults on the autism spectrum without intellectual disability. They report promising effects of this intervention in terms of improving work engagement as well as reducing behavioral problems for autistic adult.

In recent years, there have been numerous clinical trials of pharmaceutical treatments targeting core symptoms of several neurodevelopmental conditions, including ASD; however, most trials have been unsuccessful despite highly promising preclinical data. Although there are many potential reasons for these failures, the lack of adequate outcome measures and biomarkers for tracking changes in symptoms and the functioning of underlying neurobiological systems has contributed to these failures. Several of the papers in this special issue address this need for improved outcome measures and biomarkers. Brady and colleagues present data on behavioral measures that can be used to track progress in word learning, a frequent intervention target, by minimally verbal children on the autism spectrum. In their papers, Roberts et al., Beker et al., and Neuhaus et al. present data on the use of various indices of brain function derived from MEG, ERP, and EEG, respectively. These biomarkers appear to be useful for tracking improvements in neural function as well as in selecting or stratifying participants in treatment studies (e.g., into likely responders and nonresponders to a specific targeted treatment). Neuhaus et al. also report on variations in resting-state EEG as a function of various participant characteristics (e.g., sex at birth), which serves as an important reminder that an outcome measure or biomarker is likely to have a circumscribed use and needs to be matched to the characteristics of the participant sample and treatment of interest. The findings reported in these papers will be valuable in the design of future treatment studies.

Two articles present interesting frameworks for the development of new treatments for autistic individuals. In the paper by Marrus et al., the authors examine a number of risk factors for ASD, including subclinical ASD trait burden in parents, the ASD status of parents’ siblings, parent age, and transmitted autosomal molecular genetic abnormalities. Although Marrus et al. found that all four factors were associated with child outcomes, the predictive power of the set was not sufficient to be the basis of reproductive decisions (i.e., genetic counseling intervention). Their approach may have more general lessons for the field about when intervention is warranted. In their article, Gradzinski et al. argue for the value of behavioral interventions for young children at high risk for the subsequent development of ASD. Such presymptomatic interventions build on recent research identifying early signs of later ASD with the aim of preventing subsequent symptoms of ASD before they become impairing. Like Marrus et al., Gradzinski and her colleagues also outline some of the complex decisions that must be made in any effort at prevention.

Several authors in this special issue use insights from more basic research findings to suggest potential targets for, and approaches to, treatment in ASD. Patterson et al. use fMRI to interrogate the distracting effects of sensory stimuli characteristic of “noisy” naturalistic environments on the identification of emotions by individuals on the autism spectrum. They also experimentally demonstrate the beneficial effects of a priming manipulation with the participants’ own emotional facial expressions, thereby raising the possibility that such priming might be useful as a target in behavioral interventions. In their article, Cook et al. used fMRI to investigate the role of schemas in memory formation by children on the autism spectrum. They found differences in the neural systems engaged relative to normative expectations and that activation patterns correlated with parent-reported behavioral inflexibility in the children. Their findings raise the possibility that intervention targeting these memory processes may have more widespread effects on a core symptom of ASD.

These 13 papers highlight the ways in which investigators in the IDDRCs are conducting research designed to reduce the challenges that can be experienced by autistic individuals. The breadth of the approaches and areas of focus nicely demonstrate the interdisciplinary nature of the IDDRCs. Although each article is led by investigators from a particular IDDRC, many articles include as co-authors investigators at other IDDRCs and other universities, reflecting the fact that the IDDRCs function as a network and as catalysts for the nation’s translational research agenda on neurodevelopmental disabilities.

## Data Availability

N/A.

